# Positive predictive value of a single nucleotide polymorphism (SNP)‐based NIPT for aneuploidy in twins: Experience from clinical practice

**DOI:** 10.1002/pd.6262

**Published:** 2022-11-21

**Authors:** Valerie Kantor, Lihong Mo, Wendy DiNonno, Katherine Howard, Charuta C. Palsuledesai, Sheetal Parmar, Zahabiya Chithiwala, Russ Jelsema, Wenbo Xu, Herman L. Hedriana

**Affiliations:** ^1^ Natera, Inc Austin Texas USA; ^2^ University of California Davis California USA

## Abstract

**Objective:**

Twins account for approximately 1 in 30 live births in the United States. However, there are limited clinical experience studies published in noninvasive prenatal testing (NIPT) for detecting aneuploidies in twins. This study reports the performance of an SNP‐based NIPT in the largest cohort with known outcomes for high‐risk aneuploidy results.

**Method:**

This is a retrospective analysis of 18,984 results from commercial single‐nucleotide polymorphism (SNP)‐based NIPT tests performed in twins between October 2, 2017 and December 31, 2019. Follow‐up for all 211 high‐risk cases was solicited.

**Results:**

Follow‐up outcomes were obtained in 105 cases. Positive predictive values (PPVs) for high‐risk results were 88.7% (63/71, 95% Confidence Interval [CI]: 79.0%–95.0%) for trisomy 21% and 72.7% (8/11, 95% CI: 39.0%–94.0%) for trisomy 18. The results were stratified into monozygotic (MZ) and dizygotic (DZ). The PPVs in MZ were 100% for both trisomy 21 (4/4, 95% CI: 40%–100%) and trisomy 18 (1/1, 95% CI: 2.5%–100%). No trisomy 13 cases were detected in the MZ group. The PPVs in DZ were 88.1% (59/67, 95% CI: 77.8%–94.7%), 70.0% (7/10, 95% CI: 34.8%–93.3%), and 66.7% (2/3, 95% CI: 9.4%–99.2%) for trisomy 21, trisomy 18, and trisomy 13, respectively.

**Conclusion:**

The performance of SNP‐based NIPT in this large twin cohort was comparable to previously reported twin NIPT studies. SNP‐based NIPT allows for zygosity‐based PPV assessment.

## Key points

What is already known about this topic?

- Limited clinical experience has been published in noninvasive prenatal testing (NIPT) for detecting aneuploidies in twin pregnancies, despite twins accounting for about 1 in 30 live births in the United States.
- SNP‐based NIPT provides both zygosity and the individual fetal fractions in dizygotics during prenatal aneuploidy screening in twin pregnancies.

What does this study add?

- This study based on a large cohort of twin pregnancies reports positive predictive values (PPVs) for detecting common aneuploidies in pregnancies identified as high‐risk‐using SNP‐based NIPT.
- This is the first study to evaluate the PPV in DZ and MZ twins using NIPT.

## 1 INTRODUCTION

Twins account for approximately 1 in 30 live births in the United States, and compared to singletons, twins have a 4‐ to 10‐fold increased risk of perinatal complications.^1^ Recent years have demonstrated a shift towards an older maternal age at conception as well as increased utilization of assisted reproductive technology (ART), both of which are associated with higher rates of multifetal gestations.^2^ Prenatal chromosomal screening is not as accurate in twin gestation compared to singleton,^2^, ^3^ and this information should be considered as a part of counseling for twins. Historically, screening of twins has used nuchal translucency and maternal serum markers. However, serum screening is problematic in twins because different levels of biomarkers contributed by the two fetuses result in lower sensitivity when compared to singletons.^2^, ^3^, ^4^ A systematic review of first‐trimester nuchal translucency and maternal serum markers in twins has reported sensitivities of less than 88% and false positive rates of 4%–10%, indicating a relatively high need for use of invasive diagnostic testing.^5^, ^6^ Following a high‐risk screening result, current clinical practice is to recommend invasive diagnostic testing (amniocentesis or chorionic villus sampling). For invasive testing, compared to singletons, twin studies have reported a higher procedure‐related fetal loss rate.^2^ Utilizing a noninvasive screening method with high sensitivity and specificity could reduce the number of pregnant women having invasive testing, reducing both risk of pregnancy loss and medical cost.^7^, ^8^

Noninvasive prenatal testing (NIPT), which utilizes cell‐free DNA (cfDNA) present in maternal circulation, is a highly sensitive and specific method to screen for common aneuploidies (trisomy 21, trisomy 18, and trisomy 13).^9^ In singleton gestations, a sensitivity of >99%, a false positive rate (FPR) <0.02%, and a positive predictive value (PPV) of >90% have been reported for screening trisomy 21 (T21) in multiple clinical validation/experience reports.^10^, ^11^, ^12^, ^13^ Because of this excellent screening performance, since 2015, NIPT has been recommended as a first‐line screen for singleton pregnancies across professional societies for obstetrician‐gynecologists and maternal‐fetal medicine specialists.^14^, ^15^ The same recommendation was made for twins in 2020 by the International Society of Prenatal Diagnosis (ISPD).^16^ In the same year, a combined statement from the American College of Obstetrics and Gynecology (ACOG) and the Society for Maternal Fetal Medicine (SMFM) noted that NIPT screening can be performed in twins, while highlighting the importance of individual adequate fetal fraction measurements for twins. These statements cited preliminary data indicating high sensitivity of NIPT in screening for trisomy 21 in twins.

Considering these recommendations supporting NIPT in twin pregnancies, additional data regarding the accuracy of NIPT based on large cohorts of twin gestations are needed to better aid in pre‐ and post‐test counseling. The sensitivity and accuracy of NIPT in singletons have been established with large cohorts, allowing for the estimation of performance with 95% confidence interval (CI), narrower than similar estimation in twins. A 2017 meta‐analysis of NIPT use in singleton pregnancies included a total of 1963 cases of trisomy 21 and allowed for the estimation of performance with a narrow 95% confidence interval (CI) with a reported weighted pooled detection rate of 99.7% (95% CI, 99.1%–99.9%) and FPR of 0.04% (95% CI, 0.02%–0.07%).^17^ Unlike singletons, a 2021 meta‐analysis of NIPT use in twins included only 137 cases of trisomy 21 and resulted in the weighted pooled detection rate of 99.0% (95% CI, 92.0%–99.9%) and FPR of 0.02% (95% CI, 0.001%–0.43%) with accordingly broader CIs.^18^ The performance of NIPT in twins based on clinical experience from a commercial reference laboratory reported an overall PPV of 79% for the common aneuploidies with the most data available for trisomy 21.^4^

In validating a single‐nucleotide polymorphism (SNP)‐based NIPT in twin pregnancies, Norwitz et al. correctly called 100% of the 11 aneuploidy cases and for all twin pairs, correctly called zygosity and fetal sex.^1^ While several publications have shown high PPVs for SNP‐based NIPT use in singleton gestations,^10^, ^11^, ^19^ the same outcome‐based PPVs have not been described for twins. Professional societies have emphasized the importance of reporting PPVs when utilizing NIPT in clinical practice.^15^ Here, we report on the performance of an SNP‐based NIPT for twin gestations in a clinical cohort. This study represents the largest clinical cohort for twin gestations with known outcomes for aneuploidy results.

## 2 MATERIALS AND METHODS

This is a retrospective analysis of 18,984 twins who had commercial SNP‐based NIPT in the United States between October 2, 2017, and December 31, 2019 and received aneuploidy results. As a component of quality assurance, the study received an exempt classification by an Investigative Review Board (Ethical and Independent (E&I), Corte Madera, CA; ID 19040‐01). All samples were processed in a CLIA‐approved laboratory (Natera, San Carlos, CA). This methodology has been previously described.^1^ Samples for twins gestation were eligible for SNP‐based NIPT if the following criteria were met: sample was of sufficient blood volume (>13 ml); sample was drawn at a gestational age of >9 weeks; sample arrived in the laboratory <8 days from blood collection; sample was collected in a Streck tube; sample was not damaged or hemolyzed upon receipt; the twin gestation was not conceived using donor oocytes. All dizygotic (DZ) samples were screened for trisomies 21, 18, and 13. Monozygotic (MZ) samples were additionally screened for monosomy X, and if ordered, 22q11.2 deletion syndrome.

Samples were classified as a high risk when receiving a risk score >1/100. For pregnancies identified as DZ using an SNP‐based assessment, reported high‐risk scores were capped with a maximum reported risk of 7/10, 1/2, and 1/3 for trisomies 21, 18, and 13, respectively, based on an analytic PPV that was calculated from predefined test sensitivity and specificity.^1^ Once identified as MZ using an SNP‐based assessment, MZ samples were processed using the singleton algorithm with a maximum reported high risk of 9/10, 9/10, and 1/2 for trisomies 21, 18, and 13, respectively, and 1/2 for monosomy X.^20^, ^21^, ^22^ A total of 211 (211/18,984, 1.1%) cases were considered to be the high risk and were included in this study.

The follow‐up for all 211 high‐risk NIPT cases was solicited by facsimile, telephone, or a combination thereof, and then recorded in a clinical database. For each patient, up to three attempts were made to obtain outcomes. Clinical information requested included the presence or absence of ultrasound anomalies, whether diagnostic testing was performed for one or both fetuses, the methodology and results of diagnostic testing, and pregnancy outcome. Samples were then classified into the following categories: (1) true positive (TP), including whether one or both twins were affected with confirmation by prenatal or postnatal diagnostic testing or clinical evaluation at birth consisting of a dysmorphology examination for features of trisomy 21, 18, or 13; (2) suggestive, defined as samples suspected to be aneuploid based on the identification of one major or two minor ultrasound anomalies in the fetus and/or soft markers, but where diagnostic testing was not obtained; (3) lost to follow‐up (LTFU), including lack of provider response to the request, follow‐up information not known to the provider, or patient transfer; (4) false positive (FP) with confirmation of euploidy by diagnostic testing or clinical evaluation at birth; (5) vanished twin (VT); and (6) early pregnancy loss (EPL). Positive predictive value (PPV) was defined as TP/(TP + FP). We calculated two sets of PPV values. The first calculation was based on TP samples defined strictly by genetic truth or clinical evaluation, and all unconfirmed cases were excluded, referred to as the “cytogenetically confirmed PPV.” The second calculation, referred to as the “suggestive findings PPV,” considered the suggestive and EPL samples in addition to the genetically confirmed samples as TP. LTFU samples were excluded from all PPV calculations. Student *t*‐test was performed to compare the combined fetal fraction of DZ twins and the total fetal fraction of MZ twins. Grouping and statistical analysis were performed with the R statistical program (version 4.0.2).

## 3 RESULTS

Of the 18,984 twin NIPT results released in the United States between October 2, 2017, and December 31, 2019, 211 were high‐risk results for an overall positive call rate of 1.11%. Of these 211 high‐risk results, 182 cases were DZ (positive call rate of 0.96%) and 23 cases were MZ (positive call rate of 0.12%). Confirmed VTs were excluded from the cohort, bringing the total number of cases to 205. The mean and the median maternal age across the high‐risk cohort were 36 and 37 years, respectively. The mean and the median gestational age for testing were 13 weeks 4 days and 12 weeks 2 days, respectively. The mean and median maternal age were consistent in the MZ and DZ groups. While the mean gestational age was lower in the MZ group (12 weeks 4 days) versus the DZ group (13 weeks 5 days), the median differed by only 2 days. The mean maternal age of this cohort was higher compared to the mean maternal age of twin cases, which received low‐risk results during the same timeframe (not included in this study) (36.1 vs. 31.7 years). However, gestational age was the same for both high‐ and low‐risk cases (13 weeks 4 days). All demographic information are outlined in Table 1.

**TABLE 1:** Demographic information for the 205 patients with twin pregnancies included in this study

	All (*n* = 205)	MZ^a^ (*n* = 23)	DZ^a^ (*n* = 182)	T21^b^ (*n* = 154)	T18^b^ (*n* = 35)	T13^b^ (*n* = 12)
Mean maternal age ± SD (years)	36.1 ± 4.7	36.1 ± 4.5	36.1 ± 4.8	36.3 ± 4.6	35.6 ± 5.1	35.3 ± 5.8
Median maternal age (years)	37	37	37	37	36	36.5
Mean gestational age at testing ± SD (days)	95.4 ± 28.6	88.4 ± 18.8	96.3 ± 29.5	95.6 ± 27.8	95.9 ± 35.2	93.7 ± 22.0
Median gestational age at testing (days)	86	85	87	87	85	86.5

Abbreviations: MZ, monozygotic; NIPT, noninvasive prenatal testing; SD, standard deviation; DZ, dizygotic.

^a^ NIPT predicted MZ and DZ twins.

^b^ High risk for aneuploidy reported by NIPT.

**FIGURE 1:**
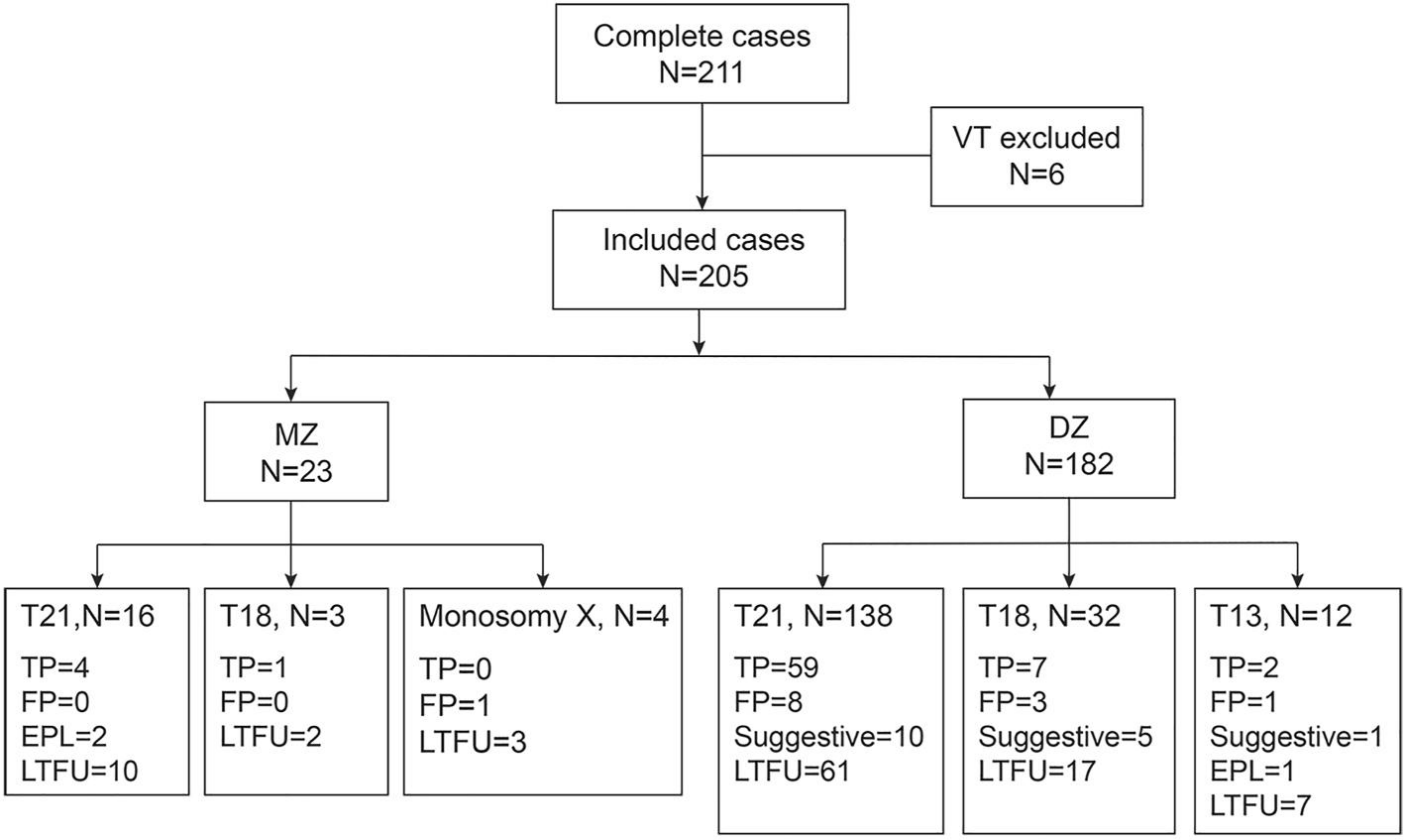
Summary of inclusion criteria and results of patients with twin pregnancies undergoing SNP‐based NIPT. EPL, early pregnancy loss; FP, false positives; LTFU, lost to follow‐up; MZ, monozygotic; *N*, number of patients; VT, vanished twin; DZ, dizygotic; TP, true positives.

A flow chart of all samples in the study cohort is presented in Figure 1. Fetal outcomes were obtained in 51.2% (105/205) of cases with a high‐risk report. Trisomy 21 was the most common high‐risk result, accounting for 75.1% of results (154/205), followed by trisomy 18 (17.1%; 35/205), and then trisomy 13 (5.9% 12/205). Additionally, four cases were called high risk for monosomy X, a condition only evaluated in MZ pregnancies; these cases were kept separate from the combined PPV calculations.

Overall, PPV was calculated for trisomy 21, trisomy 18, and trisomy 13 (Table 2). For high‐risk trisomy 21 results, the outcome was obtained in 52.6% of cases (81/154). PPV including cytogenetically confirmed cases only (cytogenetically confirmed PPV) as well as PPV including cases with suggested ultrasound findings and EPL in addition to the cytogenetically confirmed cases (suggestive findings PPV) were calculated for each condition. An estimated cytogenetically confirmed and suggestive findings PPV of 88.7% (63/71) and 90.4% (75/83), respectively, were observed for trisomy 21. Similarly, the estimated cytogenetically confirmed and suggestive findings PPVs for trisomy 18 cases were 72.7% (8/11) and 81.3% (13/16), respectively. And the estimated cytogenetically confirmed and suggestive findings PPVs for trisomy 13 were 66.7% (2/3) and 80.0% (4/5), respectively (Table 2).

**TABLE 2:** Performance of SNP‐based NIPT to screen for trisomy 21 (T21), trisomy 18 (T18) and trisomy 13 (T13)

	PPV calculation method	T21^b^	T18^b^	T13^b^
MZ^a^ twins	Cytogenetically confirmed cases (%)	100 (4/4). [40%–100%]	100 (1/1), [2.5%–100%]	‐
	Suggestive findings PPV (%)	100 (6/6), [54%–100%]	100 (1/1), [2.5%–100%]	‐
DZ^a^ twins	Cytogenetically confirmed cases (%)	88.1 (59/67), [77.8%–94.7%]	70.0 (7/10), [34.8%–93.3%]	66.7 (2/3), [9.4%–99.2%]
	Suggestive findings PPV (%)	89.6 (69/77), [80.6%–95.4%]	80.0 (12/15), [51.9%–95.7%]	80.0 (4/5), [28.4%–99.5%]
All twins (MZ + DZ)	Cytogenetically confirmed cases (%)	88.7 (63/71), [79.0%–95.0%]	72.7 (8/11), [39.0%–94.0%]	66.7 (2/3), [9.4%–99.2%]
	Suggestive findings PPV (%)	90.4 (75/83), [81.9%–95.8%]	81.3 (13/16), [54.4%–96.0%]	80.0 (4/5), [28.4%–99.5%]

*Note*: The calculations were based on 105 cases with outcomes out of 205 cases reported as a high risk. Cytogenetically confirmed PPV, consisting of only cytogenetically confirmed cases, and suggestive findings PPV, including cases with suggestive ultrasound findings and EPL in addition to the cytogenetically confirmed cases, were calculated for each condition.

Abbreviations: DZ, dizygotic; MZ, monozygotic; PPV, positive predictive value; T13, trisomy 13; T18, trisomy 18; T21, trisomy 21.

^a^ NIPT‐predicted MZ and DZ twins.

^b^ High risk for aneuploidy reported by NIPT.

### 3.1 Dizygotic (DZ)

All results in the cohort included calls for zygosity; the results were subdivided into DZ and MZ as determined by NIPT. Dizygotic results accounted for 88.8% (182/205) of the high‐risk cohort. Similar to the combined dizygotic/monozygotic cohort, the overall test performance in dizygotic cases with cytogenetic confirmation showed an estimated PPV of 88.1% for trisomy 21% and 85% for all aneuploidies. When cases with suggested findings or EPL were included with cytogenetically confirmed cases, the estimated PPV was 89.6% for trisomy 21% and 87.6% for all aneuploidies. The observational performance for trisomy 18 is highlighted in Table 2.

### 3.2 Monozygotic (MZ)

MZ results accounted for 11.2% (23/205) of the high‐risk cohort. Of these, 19/23 were high risk for trisomy with the majority (84.2%; 16/19) high risk for trisomy 21. Of those 16, follow‐up was available for 37.5% (6/16) with cytogenetically confirmed PPV and suggestive findings PPV of 100%. The remaining four MZ cases were high risk for monosomy X; due to the small number of monosomy X results, PPV calculations were not performed. A full breakdown of the MZ outcomes is included in Table S1 and the observational performance of SNP‐based NIPT is reported in Table 2.

### 3.3 Fetal fraction

Mean fetal fraction (FF) for each fetus was calculated overall and separated down into dizygotic and monozygotic values (Table S2). Previously, Hedriana et al. showed the average individual FF in DZ pregnancies was 6.4% in a combined low‐ and high‐risk cohort.^23^ While the mean FF of both fetuses in our high‐risk DZ cohort was also 6.4%, we observed a difference between the mean of the higher FF and the mean of lower FF in DZ pregnancies (7.1% vs. 5.4%). Next, we calculated mean high FF and mean low FF values for high‐risk trisomy 21, trisomy 18, and trisomy 13 results, respectively. The mean difference between FF of the two twins in pregnancies at a high risk for trisomy 21 was 1.0%. In contrast, the difference in mean FF between the two twins was greater in cases receiving high‐risk results for trisomy 18 and trisomy 13, at 2.4% and 2.5%, respectively.

## 4 DISCUSSION

In this clinical experience study utilizing SNP‐based NIPT, with 205 twin pregnancies receiving high‐risk results, cytogenetically confirmed PPVs of 88.7% and 72.7% were observed for trisomy 21 and trisomy 18, respectively. This estimate is conservative, where only cases with genetic confirmation were included in the calculation. Cases with suggestive evidence for aneuploidy (including EPLs), but without confirmation, were excluded. If we include suggestive cases, defined as suspected to be aneuploid based on the identification of one major or two minor ultrasound anomalies in the fetus and/or soft markers, and EPLs as TPs, PPVs of 90.4% and 81.3% PPV were observed. The cytogenetically confirmed PPVs in MZ twins were 100% for both trisomy 21 and trisomy 18, whereas the cytogenetically confirmed PPVs in DZ twins were 88.1% and 70.0% for trisomy 21 and trisomy 18, respectively (suggestive finding PPVs were 89.6% and 80.0% for trisomy 21 and trisomy 18, respectively). There were no cases of trisomy 13 detected in MZ twins. In DZ twins, 6.6% (12/182) cases were reported as a high risk for trisomy 13 with a PPV of 66.7%. Compared to our validation study based on 11 twin samples receiving high‐risk results, this clinical experience includes a much larger, real‐world cohort of 205 twin pregnancies receiving high‐risk results. We were able to report twin zygosity and fetal sex in all cases in this cohort. As expected, the combined FF was higher in DZ (12.9% ± 5.2%) than MZ (11.8% ± 4.4%) pregnancies. When evaluated per fetus, DZ twins had the lower FF than the average FF observed in singletons (singletons,^23^ 9.5% ± 4.1%; twin higher FF 7.1% ± 2.8%, *p* < 0.0001; twin lower FF 5.8% ± 2.4%, *p* < 0.0001). Interestingly, compared to DZ cases high risk for trisomy 21, we observed a greater FF difference in the two twins in DZ cases high risk for trisomy 18 and trisomy 13. This observation is consistent with data in singletons, where lower FF has been reported in pregnancies with trisomy 18 and trisomy 13, but not in pregnancies with trisomy 21.^24^, ^25^ As MZ twins are genetically identical, the FF per twin could not be assessed.

A recent clinical experience study using counting‐based NIPT included 422 twins with NIPT results matched with diagnostic results.^4^ Ninety‐six of these cases had positive NIPT results, and PPVs were 78.7% for trisomy 21, 84.6% for trisomy 18%, and 66.7% for trisomy 13. In contrast to this SNP‐based NIPT study, these PPVs did not discriminate by zygosity. Confirming the zygosity using SNP‐based NIPT allows for more accurate risk determination for aneuploidy risk for MZ and DZ twins.^1^

We observed that 88% (182/205) of all twin cases receiving high‐risk results in our cohort were DZ twins. Specifically, DZ twins accounted for 90% (138/154) of the high‐risk results for trisomy 21. The general population proportion of DZ and MZ pregnancies is 70% and 30%, respectively.^1^ Since DZ pregnancies involve two separate fertilization events, as compared with MZ pregnancies, which involve only one, we expect to see a per‐pregnancy aneuploidy rate in DZ that is greater than MZ; indeed, empirically observed aneuploidy rates are approximately 4 times higher in DZ pregnancies than MZ singleton pregnancies.^23^, ^26^ Our findings are consistent with the previously reported rate of trisomy 21 in MZ (6%, 11/182) and DZ (94%, 171/182) twins.^26^

The differences between PPV for MZ and DZ twins observed in this study can be explained by the complexity of incorporating two distinctive genotypes with two distinct FFs into a singular risk assessment for DZ twins. Given that their genetic signature is identical, MZ twins have a single, combined FF. As such, SNP‐based NIPT is expected to perform equally well in MZ twins as compared with singleton gestations. In DZ twins, however, the individual FF of each twin can differ by up to 9%.^23^ In a publication regarding the clinical experience of SNP‐based NIPT in singletons (*n* = 884), a range of PPV (cytogenetically confirmed PPVs and suggestive findings PPVs) of 95.7%–94.7% for trisomy 21, 93.9%–91.3% for trisomy 18, and 79.6%–67.8% for trisomy 13 was reported.^11^ In our cohort of MZ twin gestations (*n* = 19), SNP‐based NIPT resulted in cytogenetically confirmed PPVs and suggestive findings PPVs of 100% in trisomy 21 and 100% PPV in trisomy 18. No high‐risk cases of trisomy 13 were reported.

While this report presents the largest clinical experience of an SNP‐based NIPT in twins with known fetal outcomes for aneuploidy, this study has several limitations. First, the incomplete follow‐up data in high‐risk patients and no follow‐up data available in low‐risk patients precluded determination of sensitivity, specificity, and negative predictive value (NPV). Second, due to the limited number of MZ aneuploid cases, this study was underpowered to compare the PPV for MZ and DZ twins. Nonetheless, even with the exclusion of presumptive cases, the performance of the SNP‐based NIPT in this cohort was similar to PPVs reported by other twin NIPT clinical experience study.^4^ The major strength of this study is the clinical experience in the determination of individualized PPVs for MZ and DZ twins. Another strength of this study is the confirmation that the proportion of high‐risk screens for trisomy 21 is higher in DZ twins regardless of the screening method.^26^

In conclusion, this clinical experience reports high PPVs of SNP‐based NIPT in screening for trisomies 21 and 18 in twins. Given that the SNP‐based method can determine zygosity, this clinical experience demonstrates aneuploidy risk determination between MZ and DZ twins. The added complexity of incorporating two distinct genotypes with two different FF in DZ twins is hypothesized to contribute to the lower PPV for trisomy 21 in DZ twins compared with singletons. It is valuable to determine both FF and zygosity in order to provide an accurate aneuploidy risk assessment in the context of high‐risk results in twins. The performance reported here supports the use of SNP‐based NIPT as a first‐line screening test for aneuploidy in twins with the added benefit of determining zygosity, which can assist with the early detection of complications of monochorionic twin gestations, such as twin‐to‐twin transfusion syndrome.^27^, ^28^ Taking into account the lack of comprehensive follow‐up, further studies are required to determine NPV for detecting common aneuploidies in twins by SNP‐based NIPT. Furthermore, additional studies with a larger cohort are essential both to ascertain the prevalence of trisomy 13 in twins and to determine PPV following a high‐risk trisomy 13 NIPT result.

## CONFLICT OF INTEREST

Valerie Kantor, Wendy DiNonno, Katherine Howard, Charuta C. Palsuledesai, Sheetal Parmar, Russ Jelsema and Wenbo Xu are employees of Natera, Inc. with stocks or options to own stocks. Herman Hedriana is a medical advisory panel member but does not own or option to own stocks. Lihong Mo and Zahabiya Chithiwala have no conflicts.

## Supporting information

Supporting Information S1

## Data Availability

The data that support the findings of this study are available from Dr. Z. Demko (zdemko@natera.com) upon reasonable request.
