# Supplementary material for: Positive predictive value of a single nucleotide polymorphism (SNP)‐based NIPT for aneuploidy in twins: Experience from clinical practice
Source: Prenat Diagn. 2022 Nov 21;42(13):1587–93. doi: 10.1002/pd.6262 (PMC10100335; doi:10.1002/pd.6262)
Supplement: Supplementary file 1 — Supporting Information S1 [file PD-42-1587-s001.docx]

**Supporting Information**

**Title: Positive predictive value of single nucleotide polymorphism (SNP)-based NIPT for aneuploidy in twins: experience from clinical practice**

**Running Title:** PPV of SNP-based NIPT in twins from clinical experience

**Authors:** Valerie Kantor^1^, Lihong Mo^2^, Wendy DiNonno^1^, Katherine Howard^1^, Charuta C. Palsuledesai^1^, Sheetal Parmar^1^, Zahabiya Chithiwala^2^, Russ Jelsema^1^, Wenbo Xu^1^ and Herman L. Hedriana^2^

**Affiliations:**

^1^Natera, Inc, Austin, Texas, USA

^2^University of California, Davis, California, USA

Corresponding author:

Herman L. Hedriana, MD

Chief of Obstetrics, University of California Davis Health

4860 Y Street, Suite 2500

Sacramento, CA 95817

Phone: 916-734-6219

Email: [hlhedriana@ucdavis.e](mailto:hlhedriana@ucdavis.edu)du

**Supplementary Tables**

**Supplementary Table S1.**  Outcomes for 205 twin cases reported high-risk by SNP-based NIPT.

|  | **Outcome** | **T21**^‡^ | **T18**^‡^ | **T13**^‡^ | **All Aneuploidies**^‡^ |
| --- | --- | --- | --- | --- | --- |
| **MZ**^†^ **twins** | N (reported) | 16 | 3 | 0 | 19 |
|  | TP | 4 | 1 | - | 5 |
|  | Suggestive | 0 | 0 | - | 0 |
|  | FP | 0 | 0 | - | 0 |
|  | EPL | 2 | 0 | - | 2 |
|  | LTFU^§^ | 10 | 2 | - | 12 |
| **DZ**^†^ **twins** | N (twin pairs) | 138 | 32 | 12 | 182 |
|  | TP | 59 | 7 | 2 | 68 |
|  | Suggestive | 10 | 5 | 1 | 16 |
|  | FP | 8 | 3 | 1 | 12 |
|  | EPL | 0 | 0 | 1 | 1 |
|  | LTFU^§^ | 61 | 17 | 7 | 85 |
| **All twins (MZ + DZ)** | N (twin pairs) | 154 | 35 | 12 | 201^¶^ |
|  | TP | 63 | 8 | 2 | 73 |
|  | Suggestive | 10 | 5 | 1 | 16 |
|  | FP | 8 | 3 | 1 | 12 |
|  | EPL | 2 | 0 | 1 | 3 |
|  | LTFU^§^ | 71 | 19 | 7 | 184 |

Note: EPL and suggestive findings were assumed to be TPs, and both were used for the suggestive findings PPV calculation.

^†^NIPT predicted MZ and DZ twins

^‡^High-risk for aneuploidy reported by NIPT

^§^LTFU samples were excluded from PPV calculation.

^¶^Cases (N=4) with a high-risk call for Monosomy X were excluded.

T21, trisomy 21; T18, trisomy 18; T13, trisomy 13; MZ, monozygotic; DZ, dizygotic; N, number of patients; TP, true positives; FP, false positives; EPL, early pregnancy loss; LTFU, lost to follow-up; PPV, positive predictive value.

**Supplementary Table S2**. Fetal fractions (FF) observed in the 205 twin cases included in this study.

| **MZ twins** | | | | |
| --- | --- | --- | --- | --- |
| **Sample characteristics** | **Total (n=23)** | **T21 (n=16)** | **T18 (n=3)** | **T13 (n=0)** |
| Mean FF ±SD (%) | 11.8±4.4 | 12.0±4.4 | 11.5±6.0 | NA |
| Median FF (%) | 11.4 | 11.5 | 9.3 | NA |
| **DZ twins** | | | | |
| **Sample characteristics** | **Total (n=182)** | **T21 (n=138)** | **T18 (n=32)** | **T13 (n=12)** |
| Mean FF A ±SD (%) | 7.1±2.8 | 7.0±2.7 | 7.6±3.5 | 7.1±2.1 |
| Median FF A (%) | 6.5 | 6.5 | 7 | 8.1 |
| Mean FF B ±SD (%) | 5.8±2.4 | 6.0±2.3 | 5.2±2.7 | 4.6±2.0 |
| Median FF B (%) | 5.4 | 5.6 | 4.4 | 3.55 |
| Mean difference between FF A and FF B ±SD (%) | 1.4±1.8 | 1.01±1.4 | 2.4±2.8 | 2.5±1.9 |
| Median difference between FF A and FF B [range] (%) | 0.6, [0, 9.3] | 0.45, [0, 6.6] | 0.9, [0.1, 9.3] | 2.65, [0.1, 5.3] |
| Combined FF | 12.9±4.9 | 13.0±4.8 | 12.8±5.6 | 11.6±3.7 |

FF, fetal fraction; T21, trisomy 21; T18, trisomy 18; T13, trisomy 13; MZ, monozygotic; DZ, dizygotic; SD, standard deviation. For DZ twins, FF A represents a higher FF value and FF B represents the lower FF value.
